# Exercise-induced muscle damage is reduced in resistance-trained males by branched chain amino acids: a randomized, double-blind, placebo controlled study

**DOI:** 10.1186/1550-2783-9-20

**Published:** 2012-07-12

**Authors:** Glyn Howatson, Michael Hoad, Stuart Goodall, Jamie Tallent, Phillip G Bell, Duncan N French

**Affiliations:** 1 School of Life Sciences, Northumbria University, Newcastle upon Tyne, UK; 2 School of Environmental Sciences and Development, Northwest University, Potchefstroom, South Africa; 3 School of Sport Health and Applied Science, St Mary’s University College, Twickenham, UK

**Keywords:** Recovery, BCAA, Muscle damage, Resistance training

## Abstract

**Background:**

It is well documented that exercise-induced muscle damage (EIMD) decreases muscle function and causes soreness and discomfort. Branched-chain amino acid (BCAA) supplementation has been shown to increase protein synthesis and decrease muscle protein breakdown, however, the effects of BCAAs on recovery from damaging resistance training are unclear. Therefore, the aim of this study was to examine the effects of a BCAA supplementation on markers of muscle damage elicited via a sport specific bout of damaging exercise in trained volunteers.

**Methods:**

Twelve males (mean ± SD age, 23 ± 2 y; stature, 178.3 ± 3.6 cm and body mass, 79.6 ± 8.4 kg) were randomly assigned to a supplement (n = 6) or placebo (n = 6) group. The damaging exercise consisted of 100 consecutive drop-jumps. Creatine kinase (CK), maximal voluntary contraction (MVC), muscle soreness (DOMS), vertical jump (VJ), thigh circumference (TC) and calf circumference (CC) were measured as markers of muscle damage. All variables were measured immediately before the damaging exercise and at 24, 48, 72 and 96 h post-exercise.

**Results:**

A significant time effect was seen for all variables. There were significant group effects showing a reduction in CK efflux and muscle soreness in the BCAA group compared to the placebo (P<0.05). Furthermore, the recovery of MVC was greater in the BCAA group (P<0.05). The VJ, TC and CC were not different between groups.

**Conclusion:**

The present study has shown that BCAA administered before and following damaging resistance exercise reduces indices of muscle damage and accelerates recovery in resistance-trained males. It seems likely that BCAA provided greater bioavailablity of substrate to improve protein synthesis and thereby the extent of secondary muscle damage associated with strenuous resistance exercise. Clinical Trial Registration Number: NCT01529281.

## Background

Resistance exercise is a common mode of training and is considered an integral part in the athletes’ training regimen. Although many resistance exercises require both shortening and lengthening contractions, it has been well documented that exercise biased by lengthening contractions are a more powerful stimulus for neuromuscular adaptation compared to shortening contractions [1-3]. As a consequence, many athletes will routinely incorporate this exercise modality in order to maximise the potential adaptations from lengthening contractions. However, lengthening contractions, particularly when high forces are generated, precipitate temporary exercise-induced muscle damage (EIMD) that can last for several days after the initial bout [4]. This EIMD manifests as a reduction in neuromuscular function, reduced range of motion, increased muscle soreness, limb swelling and the elevation of intramuscular proteins in blood [4-6]. These signs and symptoms impair muscle function and inhibit the potential to engage in high intensity exercise on subsequent days, which is often required by athletic populations.

In an attempt to reduce the negative effects of EIMD a number of interventions have been explored; these include cold water immersions [7], antioxidant supplementation [8,9], ergogenic aids [5], non-steroidal anti-inflammatory drugs [10] and nutritional interventions [11]. These examples have shown mixed success, however one nutritional intervention, branched chain amino acids (BCAA), have shown a reasonable degree of efficacy in reducing the effects of EIMD; in the most part following strenuous endurance exercise. BCAA are a group of essential amino acids that are a key substrate for protein synthesis and recovery [12]. Furthermore, BCAA conserve muscle mass in conditions characterised by protein loss and catabolism [13] and a recent review has proposed BCAA to provide a therapeutic effect following damaging resistance exercise [14]. Indeed, studies examining recovery from heavy endurance activity [15-18] have shown evidence that BCAA are beneficial in reducing muscle damage and accelerating the recovery process.

Whilst this positive evidence is encouraging, muscle damage is far more prevalent following high intensity resistance exercise, although few studies have examined the efficacy of BCAA following damaging resistance exercise. Nosaka et al. [19] showed that amino acid supplementation (containing around 60% BCAA) was effective in reducing muscle damage and soreness when consumed immediately before and during the four recovery days that followed a damaging bout of lengthening contractions. Additionally, in a recent well-controlled example [20], muscle soreness was reduced with BCAA; however, changes in blood indices or recovery of muscle function were absent. The aforementioned studies [19,20] used untrained volunteers and an isolated muscle group, which are not wholly representative of the stimulus often encountered by many athletic populations who routinely use damaging lengthening-biased resistance exercise as a training stimulus.

Shimomura et al. [21] examined BCAA supplementation in untrained females and whilst these authors demonstrated some efficacy in reducing indices of damage in the BCAA group, the placebo control consumed carbohydrate, which has been shown to facilitate protein uptake [12,22], thus having a synergistic effect to any exogenous protein consumed following the laboratory visit. Interestingly, and in some support of this supposition, Stock et al. [23] showed that in a mixed sex group of trained participants there were no differences in damage indices between a carbohydrate versus a carbohydrate + leucine supplement. This study contradicts the general findings from other research, which may partly be attributable to a methodological difference such as providing leucine alone (and not leucine, isoleucine and valine combined). Additionally, Sharp and Pearson [24] recently examined BCAA supplementation during a resistance training programme designed to induce over-reaching. These authors showed some efficacy with BCAA supplementation in resistance-trained individuals (with the exception of creatine kinase), however, the study was not focussed on damaging exercise and/or recovery making the findings somewhat disparate. Nevertheless, the current evidence is promising and we therefore hypothesised the magnitude of EIMD in resistance-trained individuals would be lower with BCAA supplementation compared to a placebo control. Consequently, the aim of this study was to investigate the effect of BCAA supplementation on recovery from a sport-specific damaging bout of resistance exercise in trained volunteers.

## Methods

### Participants

**Figure 1:**
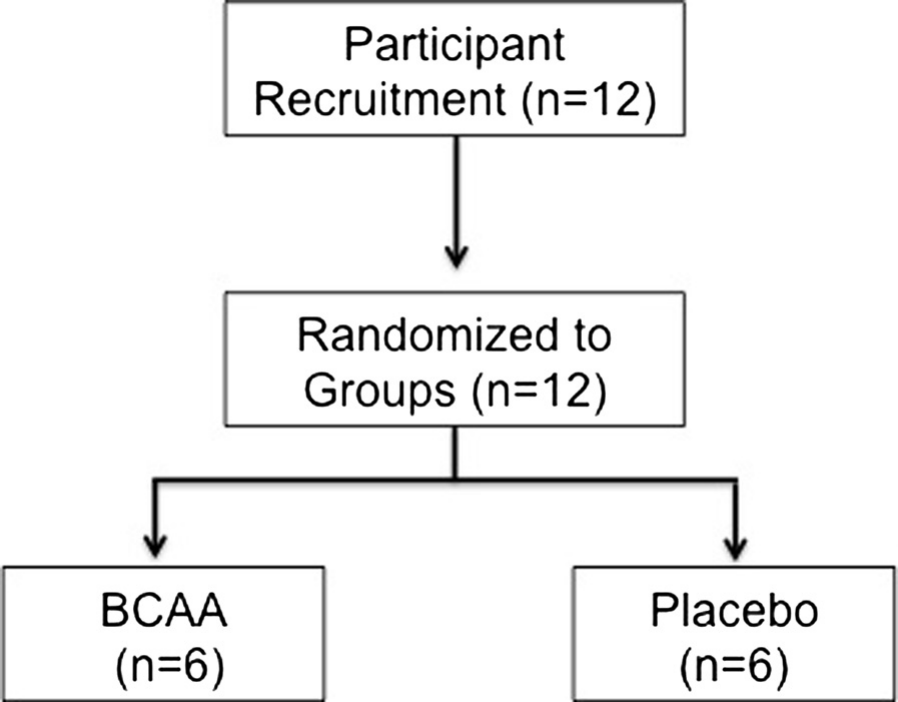
Experimental design and a flow diagram of the participants allocation to groups.

Twelve trained males who were competitive national league games players (rugby and football) and familiar with resistance training volunteered to participate (mean ± SD age, 23 ± 2 y; stature, 178.3 ± 3.6 cm; and body mass, 79.6 ± 8.4 kg). Participants engaged in specific resistance exercise at least twice per week during the competitive season. Following a health-screening questionnaire, all volunteers provided written, informed consent. Participants were randomly assigned to one of two groups, supplement or placebo, in a stratified (according to strength), double-blind fashion (Figure 1). The sample size was based on previous research examining supplementation and EIMD that had shown a significant effect [21,25]. Prior to the start of data collection all procedures were given institutional research ethics approval and subsequently registered as a clinical trial (ClinicalTrials.gov, http://www.clinicaltrials.gov, NCT01529281).

### Experimental design

The supplementation protocol followed a randomised, double-blind, placebo controlled design. The research was based around a 12 day testing period. Participants consumed either the BCAA supplement or a placebo for the duration of the study, which included a 7 day ‘loading’ phase; on day 8 the damaging exercise was performed. The criterion measures creatine kinase (CK), muscle soreness (DOMS), maximum voluntary contraction (MVC), vertical jump (VJ) and limb circumference were obtained pre-exercise and then at 24 h intervals up to 96 h post-exercise. Participants were injury free and were asked to refrain from any physical activity during the 12 day testing period and avoid taking anti-inflammatory medication, therapies and additional nutritional supplements.

### Supplementation protocol

Pre- and post-exercise supplementation lasted for a total of 12 days; this was based on previous research showing positive effects with BCAA supplementation on markers of EIMD^16^. Participants ingested 10 g, twice per day (morning and evening) of either BCAA or placebo (aspartame based artificial sweetener). The BCAA supplement (Myprotein, Cheshire, UK) contained a ratio of 2:1:1 leucine, isoleucine and valine, respectively. The BCAA and artificial sweetener were in powder form; each serving was mixed with ~300 ml of water. Artificial sweetener rather than a carbohydrate-based placebo was used to prevent a rise in insulin that may have altered protein metabolism [22]. The dosage of BCAA was based on the manufacturer’s recommendations and previous BCAA supplementation research [16,26]. Additionally, following an overnight fast, participants ingested a further 20 g bolus, 1 h pre-exercise and immediately post-exercise. In accordance with previous work [21], all participants were strongly advised to maintain regular dietary habits and avoid taking additional protein or any supplements for the duration of the study. In an attempt to control for diet, participants were asked to record food intake in the loading phase of the trial and replicate this diet as closely as possible following the damaging protocol.

### Damaging exercise protocol

Participants performed a total of 100 drop-jumps from a height of 0.6 m. Upon landing, participants were encouraged to immediately jump vertically with maximal force. Five sets of 20 drop-jumps were performed with a 10 s interval between each jump and a 2 min rest between sets. This protocol has been previously shown to cause significant elevations in muscle damage indices [19,27,28].

### Indices of muscle damage

Plasma CK was determined from an earlobe capillary blood sample. The sample was analysed immediately using an automated, dry slide photospectrometer (Reflotron Plus, Bio Stat Ltd. Stockport, UK). The normal reference ranges of plasma CK activity for this method are 24–195 IU and the intra-sample CV was<3%.

### Muscle soreness

Participants were asked to perform and hold a squat (90° knee angle) whilst they rated their perceived muscle soreness on a 200 mm visual analogue scale [5,27,29]. The scale consisted of a line from 0 mm (no pain) to 200 mm (unbearably painful).

### Maximal voluntary contraction

Isometric MVC of the participants’ dominant knee extensors was assessed using a strain gauge (MIE Medical Research Ltd., Leeds, UK). Similarly to previous work [5,11,27], participants were seated on a plinth where the strain gauge was assembled. The strain gauge was attached to the ankle, immediately above the malleoli. Each MVC was performed at a knee joint angle of 90^0^. The joint angle was assessed prior to each repetition with a goniometer (Bodycare Products, Warwickshire, UK) at the lateral condyle of the femur. MVCs were performed for 3 s with a 60 s rest between each repetition. Each participant was familiarised with the test procedure and received strong verbal encouragement for each attempt. Three MVCs were recorded and the maximum value was used for data analysis. To account for inter-subject variability, MVC was expressed as a percentage of pre-damage MVC.

### Vertical jump performance

Vertical jump (VJ) performance was assessed using the Vertec instrument (Sports Imports, Columbus Ohio). Participants performed a counter movement jump in which, on command from a standing position, they descended rapidly (to approximately a 90° knee angle) and performed a maximal vertical jump, tapping the device with the dominant arm [30]. Each participant was familiarised with the test procedure prior to the recorded efforts and received strong verbal encouragement for each attempt. Three attempts were made, each separated by 60 s, and the highest value was used for data analysis.

### Limb circumference

Mid-thigh and calf circumference was assessed as a measure of limb swelling using an anthropometric tape measure (Bodycare Products, Warwickshire, UK). Both measures were obtained with the participant in a standing position. The calf measurement was made at the widest part of the calf, whereas the mid-thigh measure was determined as the mid-point between the inguinal crease and superior aspect of the patella. Both sites were marked with semi-permanent ink to ensure consistent measurements between days [27].

### Data analysis

All data are expressed as means ± SD. Detection of differences were determined using a 2-way, repeated measures ANOVA (group, 2; time, 5). Significant interactions were followed-up using LSD *post-hoc*, pair-wise comparisons. Statistical significance was set at *P* ≤ 0.05 prior to analyses.

## Results

**Figure 2:**
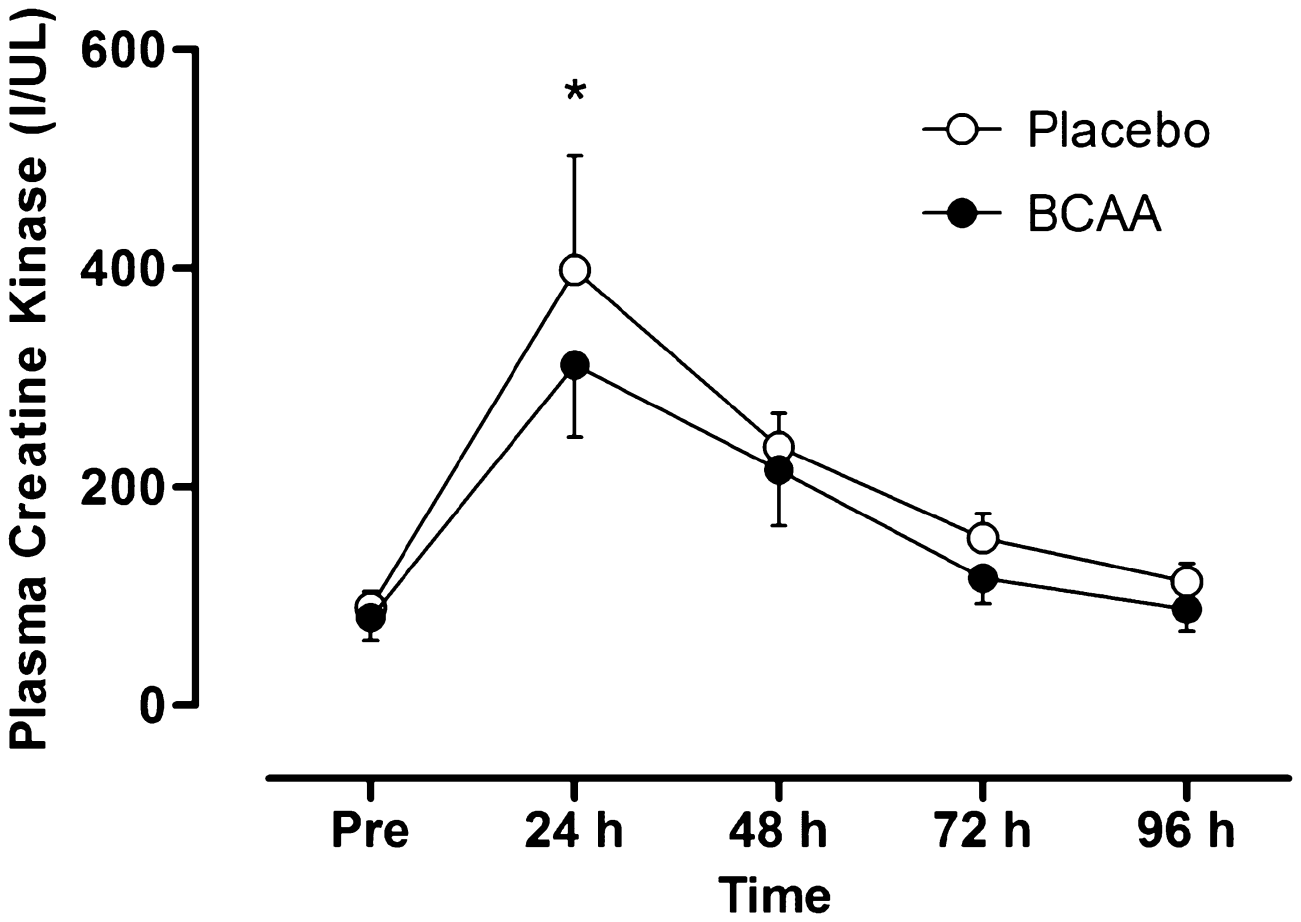
**Plasma creatine kinase concentration before and up to 96 h after the damaging bout of exercise.** * denotes a significant group effect. Values are means ± SD; N = 12.

**Figure 3:**
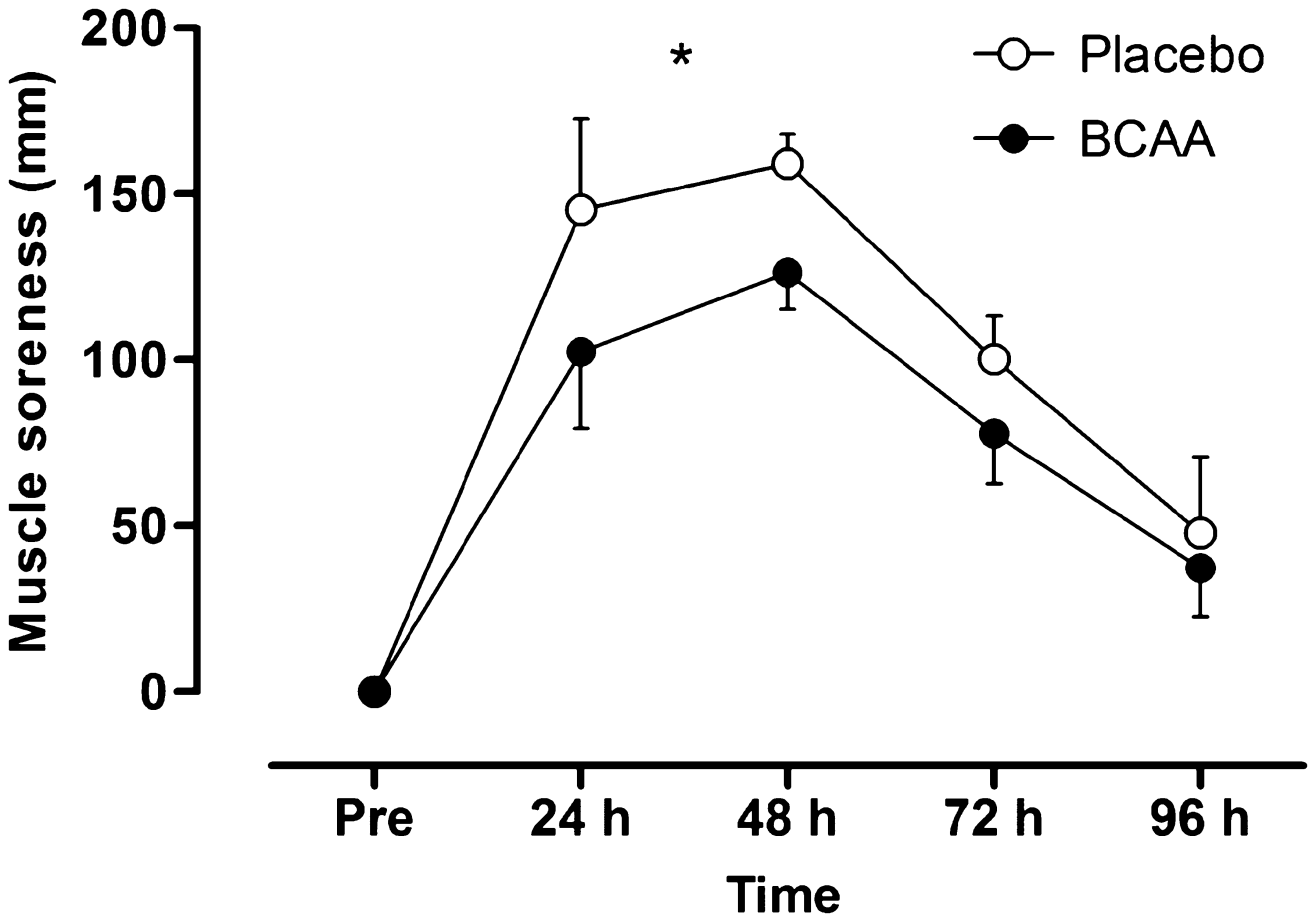
**Delayed onset muscle soreness before and up to 96 h after the damaging bout of exercise**. * denotes a significant group effect. Values are means ± SD; N = 12.

All the dependent variables showed significant time effects (*P*<0.05) demonstrating the protocol successfully induced muscle damage. CK (Figure 2) showed a significant group effect (F = 7.0, *P* = 0.024), where CK was significantly lower in the BCAA group compared to placebo. Both BCAA and placebo groups peaked at 24 h post-exercise (312 IU.L^-1^ and 398 IU.L^-1^, respectively), which equated to a 3 to 4-fold increase above baseline. Muscle soreness (Figure 3) peaked at 48 h post-exercise in both groups and showed a significant group (F = 21.3, *P* = 0.001) and interaction (F = 3.6. *P* = 0.037) effect. *Post-hoc* analysis showed that soreness was significantly lower at 24 and 48 h post-exercise in BCAA compared to control (*P*<0.05).

**Figure 4:**
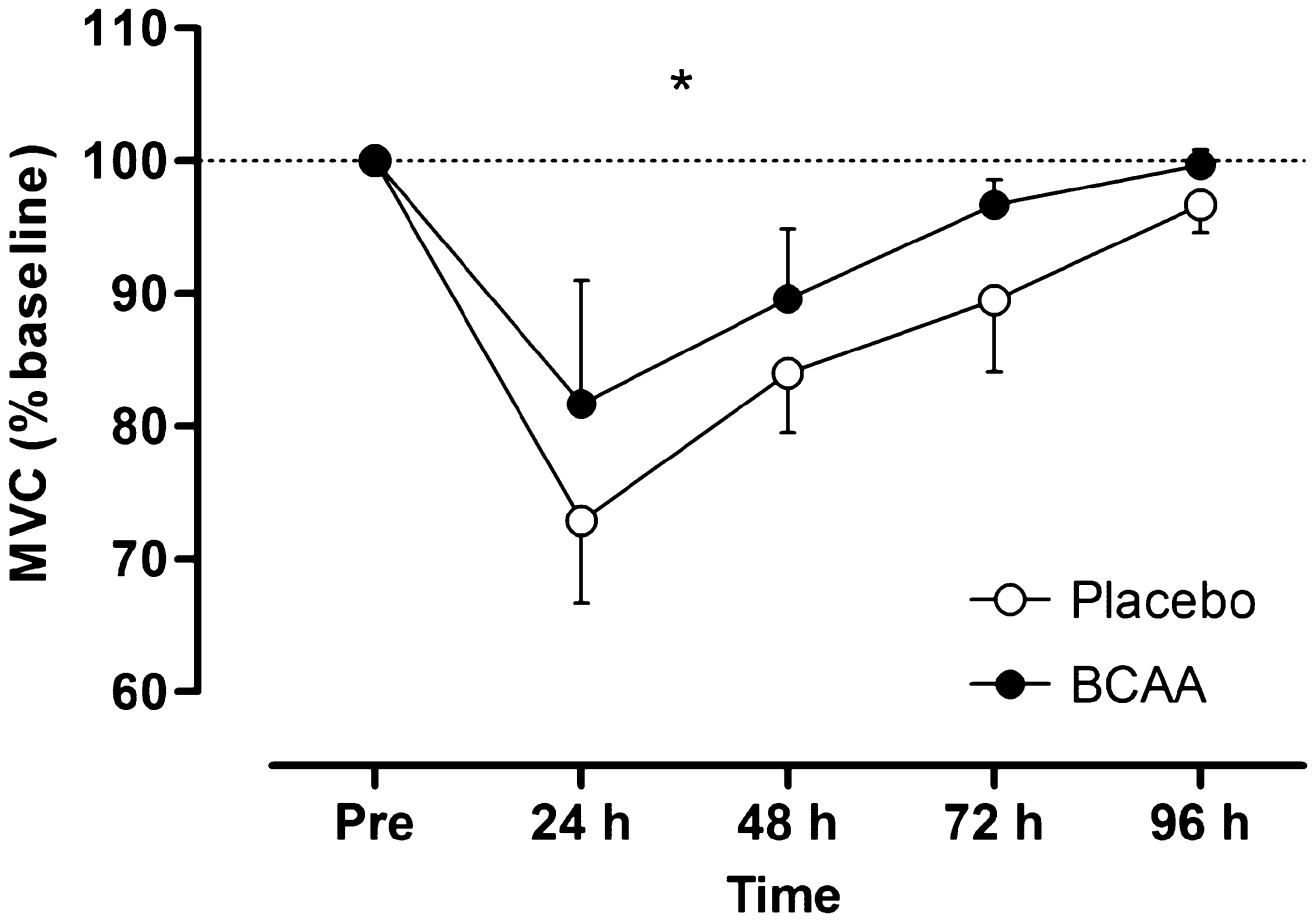
**Maximal voluntary force before and up to 96 h after the damaging bout of exercise**. * denotes a significant group effect. Values are means ± SD; N = 12.

MVC (Figure 4) showed a significant group effect (F = 9.9, *P* = 0.010) where the decrement in force was lower and recovery of force was greatest in the BCAA group. At 24 h post-exercise the BCAA and placebo groups showed a peak decrement of 18 vs. 27% below pre-exercise MVC, respectively. There were no group or interaction effects for vertical jump performance or limb girth at either the calf of thigh (Table 1).

**Table 1:** Vertical jump height, thigh and calf circumference before and up to 96 h after the damaging bout of exercise

		**Pre**			**24 h**			**48 h**			**72 h**			**96 h**		
**Vertical Jump (cm)**	**BCAA**	61.8	±	7.4	57.4	±	7.9	58.2	±	8.5	60.5	±	7.9	62.3	±	7.6
	**Placebo**	65.3	±	5.2	60.3	±	3.3	61.5	±	4.1	63.3	±	4.2	64.1	±	4.5
**Thigh Circ. (mm)**	**BCAA**	55.7	±	6.2	56.8	±	5.6	57.1	±	5.7	55.8	±	6.1	55.7	±	6.2
	**Placebo**	57.9	±	5.3	58.4	±	5.1	58.3	±	5.2	57.9	±	5.3	57.9	±	5.3
**Calf Circ. (mm)**	**BCAA**	38.1	±	1.8	38.6	±	1.5	38.8	±	1.6	38.2	±	1.8	38.1	±	1.8
	**Placebo**	37.9	±	1.3	38.3	±	1.3	38.3	±	1.4	37.9	±	1.0	37.9	±	1.0

Values are means ± SD; N = 12.

## Discussion

The initial aim of the present study was to examine the effects of BCAA supplementation on indices of muscle damage in resistance-trained volunteers. The principle findings show BCAA can reduce the negative effects of damaging exercise by attenuating CK efflux, reducing residual muscle soreness and improving recovery of muscle function to a greater extent than a placebo control.

The protocol successfully induced muscle damage, which was evident from the significant time effects for all dependent variables. This supports the efficacy of the protocol as a model to induce muscle damage in a sport specific manner [27,28]. Additionally, the data presented here support previous literature suggesting BCAA as an effective intervention to reduce the negative effects of damaging exercise [15-18] and more specifically from damaging *resistance* exercise [14,20,21]. The novel information offered by these data demonstrate that BCAA can be used as an effective intervention to ameliorate the negative effects EIMD precipitated from a sport specific damaging bout of resistance exercise in trained participants.

Creatine kinase, a surrogate index of muscle damage, is more indicative of damage or gaps in the sarcolemma and hence causing the cytosolic enzymes to ‘leak’ from the cell in to the blood [20]. However, the cell membrane is likely to have undergone some degree of lipolysis as a result of an imbalance in calcium homeostasis [4], almost certainly from the exercise insult. The damage literature often shows a high degree of inter-subject variability in CK and other cytosolic markers of EIMD, however, variability in the current study was relatively small, partly attributable to the trained status of the volunteers. The greater conditioning of these participants has almost certainly led to a repeated bout effect [31], whereby, a conditioning bout of exercise (in this case prior training) leads to a decrease in damage indices on subsequent bouts [4,31,32]. This is further supported by the low CK response seen in both groups following the exercise, when compared to the damage responses seen in untrained volunteers [19,20]. Despite this relative homogeneity, the CK response was less in the BCAA group suggesting the membrane integrity was maintained to greater extent than the placebo group. The damage response is known to be bi-phasic in nature; a primary response caused by the mechanical stress of the exercise, followed by a secondary, transient inflammatory response over the following hours and days [4]. The subsequent inflammatory response increases protein uptake necessary for use as an energy source and/or pathways responsible for cell signaling and subsequent muscle remodeling [14,33]. Although we cannot definitively support this postulate, it seems plausible that the greater bioavailability provided by BCAA facilitated this response and thereby decreased secondary damage to the muscle.

Our data concur with previous studies that show a peak in soreness at 48 h post-exercise [27,32]. Furthermore, the group effects support previous data [20,21,34] showing a reduction in muscle soreness following a damaging bout of exercise with BCAA supplementation. Although the mechanism surrounding muscle soreness following a damaging bout of exercise is not well understood, it seems likely to be related to inflammation, particularly to the connective tissue elements [35] that sensitise nociceptors in muscle and hence increase sensations of pain [36]. However, previous work [20] demonstrating a reduction in soreness following BCAA supplementation also measured the acute inflammatory response (interleukin-6, a pro-inflammatory cytokine) and showed no difference between the BCAA and placebo groups. Jackman et al. [20] suggested that the increase in food or feeding *per se,* particularly amino acids, might be related to reductions in soreness. Although this idea is somewhat speculative and has no supporting evidence or proposed mechanism, we show similar trends in our data, but it is not possible to support or refute this theory. Based on the reductions in CK, it makes the expectation tenable that the secondary damage phase is reduced by the aforementioned uptake of BCAA for protein synthesis, thus, limiting the extent of damage and hence reducing the precipitation of soreness.

Whilst there was no difference in vertical jump performance and limb girth, the most notable finding is that reductions in MVC were attenuated and recovery of MVC was accelerated following BCAA supplementation. This study demonstrated an effect on function and is in contrast to other work [20] that used untrained participants in a similar experimental design showing no benefits in the recovery of force production with BCAA. Interestingly, other studies [21,37] using non-resistance-trained student populations have shown some benefit in the recovery of muscle function. These data should be treated with caution however, as both studies [21,37] used a cross-over design which suffers the limitation of the repeated bout effect (RBE). The RBE refers to a protective effect or attenuation of damage indices when the exercise is repeated [4,31,32]. Although up to 11 weeks was given between damaging bouts, the RBE has been previously shown to accelerate the recovery of muscle function for between 6 and 9 months following the initial damaging bout [38].

It would seem that differences between our findings and those of Jackman et al. [20] might lie largely with the participant populations; Jackman et al. [20] chose untrained participants, whereas the current study recruited resistance-trained volunteers. This is evident in the group familiar with resistance exercise at 72 h (> 90% recovery of MVC) in comparison to the untrained population [20] that were only ~60% recovered at the same time point. The other obvious difference between the current investigation and previous literature is the amount of BCAA administered. Historically, previous literature [21,34] examining recovery from damaging resistance exercise has only used a single bolus of ~5 g BCAA, finding small positive effects, particularly on muscle soreness. Interestingly, Jackman et al. [20] fed participants considerably more BCAA than this previous work, consisting of 88 g in total over the test period (with no loading phase), whereas the present study gave 280 g total over the test period. Our supplementation procedure included a 7 day loading phase (20 g per day) and 20 g per day during the subsequent recovery phase. Furthermore, we provided a 20 g dose immediately before and after the bout of exercise, which is when the biggest discrepancy in BCAA feeding occurred between studies. Previous work [39] has shown that timing of a protein based recovery strategy is important and immediately following a damaging bout of exercise can be most beneficial in accelerating recovery. Whist Jackman et al. [20] did supplement with BCAA after the damaging bout, there was a delay of at least 1 h that may also account for the positive effect found in the present study, which fed immediately after the bout of damaging exercise. Previous work [40] showed BCAA to rise in plasma within 15 min and peak 30 min after ingestion, which means the bioavailability of BCAA post-exercise in our investigation was at least 1 h earlier than that of Jackman et al. [20]; therefore, it seems plausible that early feeding post-damaging exercise increased the efficacy of the intervention. This is somewhat conjectural and would serve as an interesting question for future research to ascertain the optimal strategy for BCAA supplementation.

Regardless of whether the loading phase and timing of the supplementation post-exercise was effective in increasing the bioavailability of BCAA, there is still a stark difference in the total supplementation volume (88 vs. 140 g). The larger quantity of BCAA we provided might partly account for the difference between studies in damage indices (MVC and CK). We based our supplementation regimen on previous work that showed a positive effect [16,26] and propose that positive effects beyond attenuation of muscle soreness (i.e., recovery of muscle function) may need a more immediate bioavailability and greater quantity of BCAA than those used previously.

There are two limitations from the study, which need to be acknowledged. Firstly the lack of specific dietary control might have led to discrepancies in caloric and, more specifically, protein ingestion between the groups. Although we attempted to control this by asking participants to record food intake during the loading phase and replicate this following the damaging exercise, an approach that has been previous used [11,21], there was no specific control between groups. Conceivably discrepancies in protein intake can affect the bioavailability of the substrate and hence affect protein turnover and ultimately influence the outcome of these data. The second limitation is that we used an artificial sweetener with little or no calorific value was used, which will certainly alter the energy balance by around 80 kcal/day, and may be problematic if the placebo group were in energy deficit, but based on the food record sheets this does not seem likely. Although the current investigation has a good degree of external validity, future research might like to consider more rigorous dietary control measures such as; 1) asking participants to weigh food and accurately log food intake; or 2) providing a pre-determined menu for the participants to ensure no discrepancies between and within groups, although this still relies on participant adherence outside the laboratory. Finally, 3) although difficult to facilitate, participants could be housed in an environment where dietary behavior can be imposed and thereby strictly controlled.

In summary, these data offer novel information on the application of BCAA supplementation. A 20 g/day supplementation regimen administered 7 days prior to (with additional 20 g *immediately* before and following the damaging exercise) and for 4 days after a damaging bout of eccentric biased exercise reduced soreness and the plasma level of intramuscular enzymes. Most importantly, BCAA attenuated reductions in muscle function and accelerated recovery post-exercise in a resistance-trained population.

## Competing interests

The authors declare that they have no competing interests.

## Authors’ contributions

GH, as the principal investigator, contributed to conception and design of the experiment, data collection and analysis, data interpretation, manuscript draft and the editorial process. MH, as a post-graduate student, was responsible for conception, participant recruitment, data collection, initial data analysis, interpretation and initial drafting of the manuscript. SG contributed to data interpretation, data presentation and manuscript drafting and editing. JT, PGB, DNF contributed to data analysis, data interpretation and manuscript editing. All authors approved the final version of the manuscript.
